# A Curious Case

**Published:** 1894-08

**Authors:** 


### A Curious Case.

The history of an interesting and unique case of suppuration in connection with the lower jaw is recorded in the April issue of the *Dental Record*, by Mr. F. Rooke. A patient, aged 19, was admitted into hospital for a well-marked swelling extending over the right side of the cheek and neck. This condition was attended with a good deal of trismus, the temperature being 103° F. Fluctuation was distinct, and the parts extremely painful. Under an anæsthetic an examination of the mouth was made, and two carious molars on the right side removed, this operation being followed by a profuse discharge of offensive pus. Two days subsequently the temperature had fallen to 99° F; the swelling had greatly subsided, but a good deal of pain still existed, while an opening on the cheek appeared inevitable. This abscess was at the end of eight days opened, with a result that the pain felt considerably easier; the suppuration still continued to spread forwards and showed signs of pointing again midway between the angle and symphysis. In the act of irrigating the original sinus and squeezing out the pus, a *melon seed* made its appearance, and from this point the symptoms took a favorable turn, the patient making a speedy recovery. *Journal of the British Dental Association.*

